# A Minimal Model of Metabolism-Based Chemotaxis

**DOI:** 10.1371/journal.pcbi.1001004

**Published:** 2010-12-02

**Authors:** Matthew D. Egbert, Xabier E. Barandiaran, Ezequiel A. Di Paolo

**Affiliations:** 1 Centre for Computational Neuroscience and Robotics, University of Sussex, Brighton, United Kingdom; 2 Ikerbasque: Basque Foundation for Science, Department of Logic and Philosophy of Science, University of the Basque Country, San Sebastián, Spain; University of Illinois at Urbana-Champaign, United States of America

## Abstract

Since the pioneering work by Julius Adler in the 1960's, bacterial chemotaxis has been predominantly studied as metabolism-independent. All available simulation models of bacterial chemotaxis endorse this assumption. Recent studies have shown, however, that many metabolism-dependent chemotactic patterns occur in bacteria. We hereby present the simplest artificial protocell model capable of performing metabolism-based chemotaxis. The model serves as a proof of concept to show how even the simplest metabolism can sustain chemotactic patterns of varying sophistication. It also reproduces a set of phenomena that have recently attracted attention on bacterial chemotaxis and provides insights about alternative mechanisms that could instantiate them. We conclude that relaxing the metabolism-independent assumption provides important theoretical advances, forces us to rethink some established pre-conceptions and may help us better understand unexplored and poorly understood aspects of bacterial chemotaxis.

## Author Summary

Traditionally, bacterial chemotaxis has been treated as metabolism-independent. Under this assumption, dedicated chemotaxis signalling pathways operate independently of metabolic processes. There is however, in various strains of bacteria, growing evidence of metabolism-*dependent* chemotaxis where metabolism modulates behavior. In this vein, we present the first model of metabolism-based chemotaxis that accomplishes chemotaxis without transmembrane receptors or signal transduction proteins, through the direct modulation of flagellar rotation by metabolite concentrations. The minimal model recreates chemotactic patterns found in bacteria, including: 1) chemotaxis towards metabolic resources and 2) away from metabolic inhibitors, 3) inhibition of chemotaxis in the presence of abundant resources, 4) cessation of chemotaxis to a resource due to inhibition of the metabolism of that resource, 5) sensitivity to metabolic and behavioral history and 6) integration of simultaneous complex environmental “stimuli”. The model demonstrates the substantial adaptability provided by the simple metabolism-based mechanism in the form of an ongoing, contextualized and integrative evaluation of the environment. Fumarate is identified as possibly playing a role in metabolism-based chemotaxis in bacteria, and some consequences of relaxing the metabolism-independent assumption are considered, causing us to reconsider the categorization of environmental compounds into “attractants” or “repellents” based solely on their binding properties.

## Introduction

Bacterial chemotaxis is one of the best known examples of adaptive unicellular motility. In particular, the mechanisms underlying chemotaxis in *Escherichia coli* have been studied in detail for the last 40 years (for recent, comprehensive reviews see e.g., [1]–[3]). Since the work of pioneers such as Adler [4], [5], Berg [6], Macnab [7], and Spudich [8], considerable advances continue to be made concerning the molecular structure of motors [9], [10], the structure of transmembrane receptors and their collective dynamics [11], [12] and the details of a two component signal transduction system [13] that mediates between sensors and motors [14]. Computer simulations of the underlying biochemical processes have helped to support and clarify the current model of chemotaxis mechanisms [15], [16].

In this paper we explore, by means of minimal simulation models, the widespread assumption that the mechanisms of bacterial chemotaxis operate independently of metabolism [4]. In this prevailing ‘metabolism-independent’ view, the behavior generating mechanisms such as sensors, transduction pathways, flagella, etc., are the product of metabolism, but their ongoing, short-term activity is not subsequently influenced by metabolism. In other words, in the short term, behavior is not sensitive to changes in the metabolism.

In contrast to this view is metabolism-dependent chemotaxis, where the metabolism has an ongoing influence upon behavior. The concept dates back at least as far as 1953 [17], but fell out of favour when Adler demonstrated that in *E. coli*, metabolism of a reactant is neither necessary nor sufficient for taxis [4]. Interest was rekindled in a 1983 review of the role of the proton motive force in taxis mechanisms [18] and there is growing evidence for metabolism-sensitive chemotaxis in *Azospirillum brasilense* [19], *E. coli* [20], and other bacteria [21], suggesting that *metabolism-dependent* chemotaxis might be more prevalent that previously assumed (see [22] for a recent review of metabolism-dependent ‘energy taxis’).

In this paper, we clarify the distinction between the different relationships between metabolism, chemotaxis and its generative mechanisms and we demonstrate how a metabolism-based chemotaxis mechanism is capable of generating several phenomena observed in bacteria. Our model demonstrates the substantial adaptability provided by the simple metabolism-based mechanism in the form of an ongoing, contextualized and integrative evaluation of the environment. We conclude by discussing this adaptability, the possibility of fumarate playing a role in metabolism-based chemotaxis in bacteria, and some consequences of relaxing the metabolism-independent assumption.

To avoid misunderstanding, we shall clarify two different usages of the term “adaptive” or “adaptation” in this paper. The first usage is that of “organismic or physiological adaptation”, meaning the capacity of an organism to homeostatically maintain essential variables (e.g., temperature, pH level, etc.) within viability boundaries, or to maximize or minimize their value (e.g., maximize the amount of available food or minimize exposure to a toxin). Regulated motility is a widespread means for achieving this type of adaptation. For instance, an organism can maintain a stable level of sucrose by moving to sucrose rich environments or moving away from them, or maximize the amount of light by moving to brighter areas, etc. This meaning of adaptivity is well established in biology and complex systems, (see e.g., [23]). The second sense of adaptation is that of “sensory adaptation”, used specifically by the bacterial chemotaxis research community to mean the capacity of transmembrane receptors to maintain the same degree of sensory sensitivity in an extremely wide range of base-stimulus levels [24]. Bacterial chemotaxis can be adaptive (in the first, physiological / organismic meaning of adaptation) without any sensory adaptation (second meaning) taking place, provided that mechanisms other than sensory adaptation are capable of guiding behavior efficiently. So, what matters for a behavior to be adaptive or conducive to the stability (or maximization of a given variable) is not the type of behavior generating mechanisms (e.g., transmembrane signal transduction proteins), nor the dynamics of subcomponents of such mechanisms, but just the global resulting pattern of behavior. Unless stated otherwise, in this paper the word adaptation is used in the first, organismic sense.

We wish to include one additional terminological clarification: we consider a behavior to be *chemotactic* if there is, in general, an effective overall behavior that results in approach towards specific chemical environments, irrespective of the mechanism that generates the behavior.

### Chemotaxis through running and tumbling

**Figure 1:**
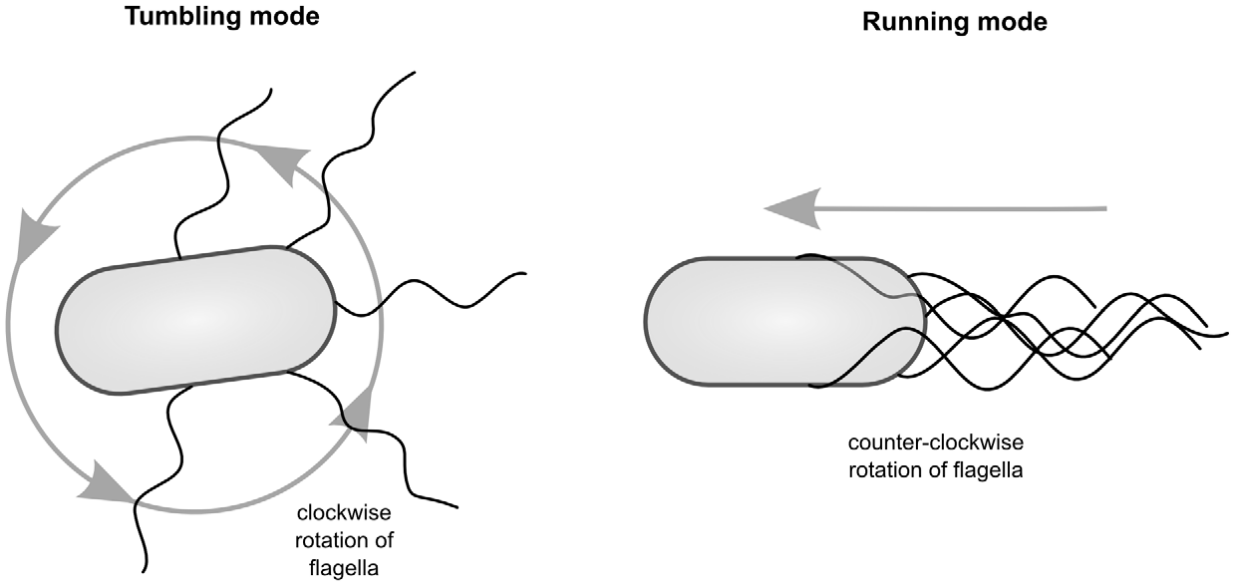
Tumbling and running modes associated with *Escherichia coli* and *Salmonella typhimurium*. CW rotation results in a random re-orientation for the bacterium, but CCW rotation of flagellar motors produces an approximately straight-line motion.

Behavioral analysis of chemotaxis in *E. coli* has shown that up-gradient or down-gradient directional movement is achieved through the combination of two basic types of movements, tumbling and running (see Figure 1). These two behaviors are both achieved through the rotation of flagella. Rotating the flagella in one direction (counter-clockwise) results in a directed motion of the bacterium called ‘running’ while brief periods of rotation in the other direction cause ‘tumbling’, the production of a more-or-less random new orientation. Many swimming bacteria make use of similar patterns, alternating between a straight motion mode and a random change of direction [25]. Bacteria combine these movements in such a way as to produce a stochastic chemotactic behavior. How do they accomplish this? In some species this is relatively well understood. For instance, in *E. coli*, a two component signal transduction system [13] compares current and past concentrations of attractants and causes the bacterium to run if the concentration of an attractant is increasing and tumble otherwise (see e.g. [14]). This general strategy is sometimes known as ‘adaptive gradient climbing’ because it is capable of adapting to a wide range of concentrations, climbing gradients whether the local concentration is very low or very high.

This is not the only way that these basic-movements can be modulated to produce chemotaxis. What we have called the *selective-stopping* strategy (also called ‘inverted response’ elsewhere [26]) consists of a combination of running and tumbling to perform a random walk until the relevant concentrations are high at which point the tumbling motion dominates and the bacterium more or less tumbles in-place.

### Metabolism-independent chemotaxis

**Figure 2:**
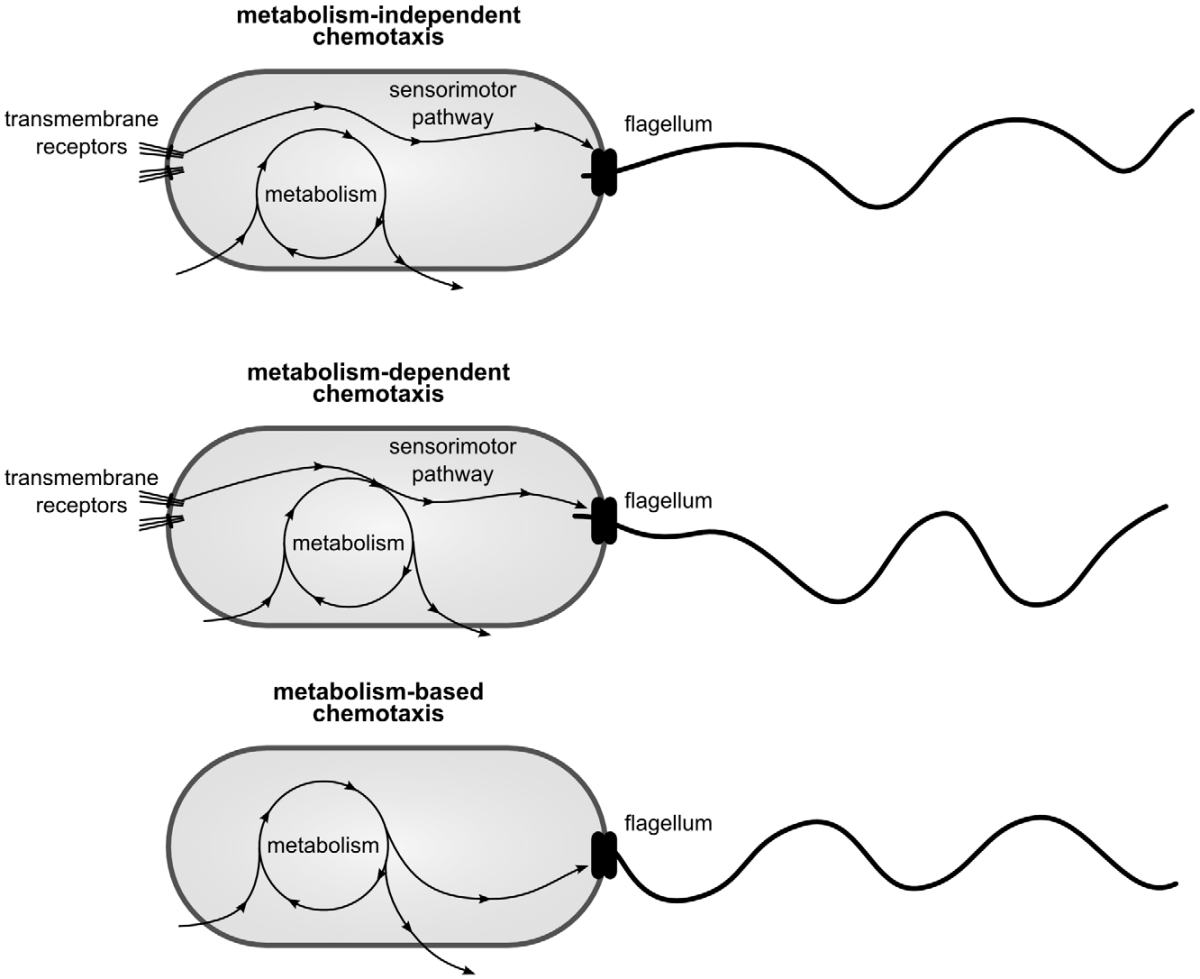
Differences between metabolism-dependent, metabolism-independent and metabolism-based chemotaxis. Arrows indicate *only* short-term dynamical influence between processes.

Regardless of which chemotactic strategy is employed, it is important to identify its *sensitivity*—what is it responding *to*? The commonly accepted view is that chemotaxis mechanisms are only responsive to the concentration of attractant chemicals in the local environment of the bacterium and are not influenced by the current state of the bacterium's metabolism. This view is known as metabolism-independent chemotaxis, for while the metabolism produces the mechanisms of sensitivity, transduction and response, the behavior of the bacterium is not influenced by the ongoing dynamics of the metabolism (see Figure 2 top). In metabolism-independent chemotaxis, it is not the effect of the attractant upon the metabolism that causes it to move towards it, it is simply the way the attractant excites the sensors. Adler, in his seminal 1969 paper, showed seemingly compelling evidence for this view of bacterial chemotaxis, providing evidence in support of the following (taken from [4]).

1. Some chemicals that are extensively metabolized fail to attract bacteria.
2. Non-metabolizable chemicals act as attractants.
3. Chemicals attract bacteria even in the presence of a metabolizable chemical.
4. Compounds that are closely related in structure compete with each other as attractants but not with structurally unrelated compounds.
5. There are mutants which fail to carry out chemotaxis to certain attractants but are still able to metabolize them.

“[R]esults show” Adler concluded “that extensive metabolism of the attractants is not required, or sufficient, for chemotaxis” [4, p.1596]. After this evidence was presented the metabolism-independent nature of *E. coli* chemotaxis became a generally accepted fact. As Alexandre and Zhulin note: “From that time on, research focused on the metabolism-independent information flow from membrane receptors to flagellar motors.” [21, p.4681]. But this assumption has recently been challenged and the consequences for the study of chemotaxis are yet to be fully disclosed.

### Evidence for metabolism-dependent chemotaxis

Despite the predominance of metabolism-independent research on bacterial chemotaxis, there is evidence of different types of metabolism mediated chemotaxis. Metabolism-*dependent* chemotaxis involves an ongoing influence of the metabolism upon the chemotaxis mechanism (see Figure 2 middle). This introduces the potential for a sensitivity to the effects of environmental phenomena upon the metabolism. In *A. brasilense*, metabolism-dependent chemotaxis has been systematically studied and is considered as the dominant behavioral strategy [19], [21]. The following behavioral phenomena have been well-established [21]:

1. nonmetabolizable analogues of metabolizable attractants are not attractants,
2. inhibition of the metabolism of a chemical attractant completely abolishes chemotaxis to and only to this attractant, and
3. the presence of another metabolizable chemical (exogenous or endogenous) prevents chemotaxis to all attractants studied (in *A. brasilense*, there is a direct correlation between the efficiency of a chemical as a growth substrate and as a chemoeffector).

Similar metabolism-dependent chemotactic phenomena have also been found in other bacteria species like *Pseudomonas putida* [27] and *Rhodobacter sphaeroides* [28], [29].

Evidence of metabolism-dependent chemotaxis has also been found in the same species that was studied by Adler, *E. coli* [20]. Adler found that although glycerol is extensively metabolized by *E. coli*, it is *not* found to act as an attractant. As such, the case of glycerol seemed to provide supporting evidence in favor of metabolism-independent chemotaxis, and so argued Adler. Interestingly, in apparent contradiction to Adler's findings, Zhulin and collaborators observed chemotaxis towards similar levels of glycerol as studied by Adler [30, p.3199].

Evidence of metabolism-dependent chemotaxis in *E. coli* has also been found for proline and succinate metabolic substrates [21]. In addition, chemotaxis to oxygen (aerotaxis) in *E. coli* [20] has been shown to depend on metabolism (i.e., reduction of oxygen is required for aerotaxis) together with redox gradient climbing [31]. These type of taxis has been termed “energy taxis” meaning that bacterial movement is sensitive to the energy production (generally by the electron transport system having modulatory effects over CheA phosphorilation, but other mechanisms have also been proposed) (for recent reviews of energy taxis, see e.g., [22], [32]).

The results described in this section all suggest that metabolism-dependent chemotaxis might be more widespread than previously thought.

### Simulation models of bacterial chemotaxis

The biochemical organization of bacteria is astonishingly complex and difficult to model. One approach is to assume some sort of functional decomposition and to study sub-systems separately. Dennis Bray and his group have achieved some impressive results along these lines [15], [16], [33]. Their model of *E. coli*'s chemotactic behavior includes molecular level details of the membranes, signal transducers, Brownian motion of molecules within the cytoplasm and a number of genetic details. Their approach has achieved unprecedented levels of predictability and empirical accuracy. However, despite the promising results, some aspects of *E. coli* chemotaxis remain elusive. For instance, how is it possible for a small number of types of sensor (5) to cause an appropriate response to a large number of attractants and repellents (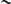
50)? It is possible that the elusiveness of these and other aspects of bacterial chemotaxis may not be a question of lack of mechanistic detail in the simulations themselves but may relate to the long-standing assumption that chemotaxis is fundamentally a metabolism-independent phenomenon.

Using a different approach, Goldstein and Soyer artificially evolved metabolism-independent chemotactic pathways (abstracting away the sensory and motor details) [26]. Chemical pathways partially reproducing a gradient climbing response could only evolve under special conditions and for a limited range of basal levels of stimuli. Contrarily, they found that the selective-stopping strategy (tumble in place if resources are high, otherwise perform a random walk) was comparatively easy to evolve. The resulting selective-stopping strategy is simpler, yet robust and efficient. This strategy is also found in different types of bacteria particularly in those performing metabolism-dependent chemotaxis—like that of *Rhodobacter sphaeroides* [34]—and in *E. coli* when “normal” transduction pathways are knocked down [35]. Goldstein and Soyer found very simple chemical pathways capable of generating such response patterns. They speculate that “non-adaptive dynamics [i.e. the selective-stopping strategy] could even be achieved without any signaling proteins; a small molecule, that is a by-product of metabolism or is taken into the cell via a transporter, could directly regulate tumbling probability of the cell”, [26, p.5].

In order to further develop this hypothesis, we start from an idealized minimal metabolism and make it support and generate chemotactic behavior. Our model provides a proof of concept of how a minimal metabolism could already support experimentally observed metabolism-dependent chemotactic patterns. The model displays the response patterns shown by Goldstein and Soyer and, in addition, it reproduces some of the metabolism-dependent phenomena described by Alexander and Zhulin (2001) [21].

### Summary of key concepts

To summarize, *metabolism-independent* chemotaxis refers to types of chemotaxis where the chemotaxis generating mechanisms operate rather independently from metabolism. Flagellar rotation is only influenced by chemical pathways that are independent from the metabolic network and only modulated by transmembrane receptor activity (see Figure 2 top). In *metabolism-dependent* chemotaxis, the chemical pathway that mediates transmembrane receptors and flagellar rotation is influenced by or coupled with metabolic pathways and processes (like the electron transport system) with the result of a metabolism-sensitive behavior (see Figure 2 middle). A third possibility is *metabolism-based* chemotaxis, in which metabolism itself directly modulates behavior. In this case, there are neither specialized sensors (like transmembrane proteins) nor specialized and dynamically decoupled chemical pathways (see Figure 2 bottom).

Most of the available simulation models of chemotaxis are of metabolism-independent scenarios, there are no models of metabolism-dependent or metabolism-based chemotaxis. In the next section, we introduce a first model of metabolism-based chemotaxis to study its chemotactic potential and some theoretical implications (the likelihood of the specific mechanisms in play and experimental support remain out of the scope of this paper).

## Methods

### A minimal model of metabolism-based behavior

The first step to create a minimal metabolism-based chemotactic agent is to distill and justify what counts as a minimal model of metabolism. We do not pretend to settle this issue here but we provide a general context that justifies the assumptions built into the model.

It is generally accepted that life depends upon energetic and material resources in its ongoing self-(re)-production, self-maintenance and growth. Energy and matter flow through living systems, maintaining their biophysical and chemo-dynamic organization. These two flows are coupled: energy is used to transform materials into structures that harness energy to perform further material transformations [36]–[38]. In concrete terms, the energetic flow through the system depends upon the existence of catalysts which in turn depends upon the flow of energy through the system. This autocatalytic closure of chemical reactions has long been argued to constitute the core or essence of living organization, exemplified in metabolism [38]–[41]—see [42], [43] for recent reviews and, more generally, see [44], [45] for the central role played by metabolism in grounding biological agency and adaptivity. This metabolic organization stands far from thermodynamic equilibrium. Energy and matter are lost as heat and waste, requiring the continued acquisition of new resources.

Abstracting away from the particularities of different metabolic networks, the following three key features remain essential to characterize metabolism:

1. A flow of matter through the system.
2. A flow of energy through the system.
3. A dissipative (or degrading) organization that in the prolonged absence of sufficient resources (energetic or material) ceases to exist.

**Figure 3:**
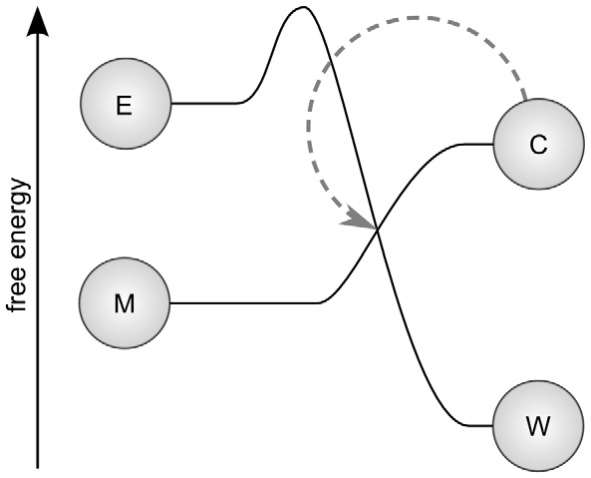
Conceptualization of a minimal metabolism. An exergonic reaction (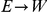) is coupled to an endergonic material transforming reaction (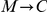) such that system components are produced (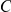) that catalyze those reactions.

One of the simplest systems that has these features consists of the following autocatalytic reaction.
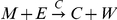
where 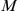 and 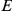 represent material and energetic resources respectively, 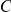 is a constituent or catalyst molecule and 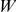 is low-energy waste. The 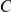 above the arrow represents catalysis of this reaction by 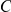. This system is illustrated in Figure 3, which also indicates the relative free energies of the reactants on the vertical axis. One can conceive of this reaction, according to the free-energies, as an exergonic reaction (in which energy is released as 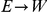) that is coupled to (and drives) the endergonic reaction of 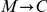. Real metabolisms, of course, have many intermediate steps in the production of enzymes which complicate the system. We assume that such intermediate steps can be justifiably abstracted away to illustrate a minimal instance of metabolism-based chemotaxis, taking the equation above to represent the higher order chemical dynamics of a whole metabolic network.

The 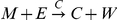 autocatalysis and modulation of flagellar rotation by the concentration of 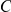 is indeed a simple mechanism. In theory, this simplicity could be taken even further. For instance, the energetic and material resources could come from a single molecule, 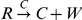. We chose to keep these resources separate so that we could investigate issues related to integration and to explore more complex environments (see Experiments 2–6). For those experiments where 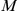 and 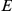 are distributed in the same spatial distribution, the reaction 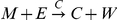 and the simpler reaction 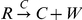 play qualitatively equivalent roles and behavioral results are identical (results not shown). We chose to maintain the same core reaction of 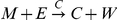 throughout this paper for simplicity and parsimony.

The waste particle 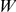 also does not play a critical role in the dynamics that we have observed. And in fact, the autocatalysis is not strictly necessary to produce chemotactic behavior (although it has dynamic consequences). A chemical reaction as simple as 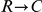 could have captured a large portion of the behavioral dynamics we have described here. However, we set out with the motivation of exploring metabolism-based chemotaxis. As we described earlier in this section, a metabolism requires the channeling of energy by enzymes into reactions that produce more of those enzymes. We have tried to capture that essential relationship in the minimal reaction 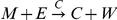.

The following subsections describe the details of the modeling environment, how the above core reaction (and some variations) are implemented to model a minimal metabolism and how a simple coupling of this metabolism to an abstraction of the flagellar machinery generates chemotactic behavior.

### Model

The model consists of a two-dimensional environment, containing resource gradients and simulated bacteria. Each bacterium has a position, orientation and velocity as well as a metabolism, which is represented by a set of chemical concentrations. The concentrations of these chemicals are updated each iteration through numerical integration of the differential equations that represent ongoing chemical reactions in the metabolism as well as degradation of metabolites and transport of ‘resource’ molecules from the immediate environment of the simulated bacteria into their interior.

Each bacterium is always either ‘running’ (moving in a straight line) or ‘tumbling’ (changing its orientation randomly). The probability of tumbling is directly proportional to the concentration of 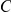, the autocatalytic product of the metabolism. This metabolism-based behavioral mechanism causes the bacterium to remain still when the metabolic rate is sufficiently high and to run when its metabolism is not operating above a threshold rate. The simulated environment is 200 units square. Bacteria trying to move out of this area are prevented from doing so as if running into a wall. Details of the reactions, the behavioral mechanisms and the environment are given below.

### Chemical reactions

The autocatalytic reaction that constitutes metabolism is more explicitly described by the following reaction equations that include the intermediate stage where the catalyst, 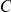, is bound to one of its substrates, 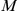, forming 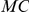.
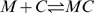
(1)

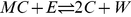
(2)Two other processes influence the concentration of the metabolic reactants. The first is the degradation of reactants 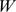 and 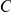 into non-reactive products. These chemicals are removed from the simulation at rates specified in Table 1. The second is the influence of the local environmental chemical concentrations upon the concentration of reactants within the simulated bacteria (described below).

**Table 1:** Constants.

**Free energies**			
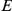 , 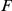	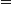	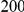	
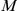 , 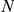	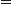	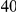	
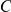	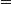	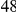	
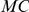 , 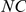	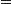	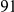	
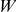	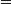	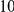	
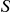 , 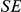	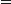	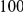	
**Reaction activation energies**			
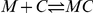	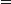	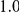	(Reaction 1)
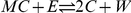	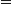	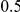	(Reaction 2)
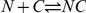	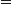	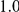	(Reaction 3)
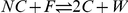	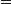	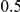	(Reaction 4)
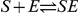	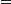	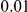	(Reaction 5)
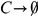	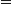	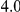	
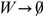	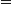	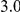	
**Reaction rate constants**			
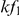 , 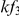	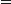	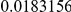	
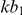 , 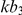	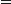	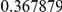	
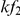 , 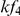	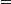	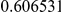	
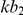 , 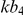	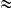	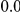	
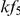	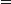	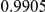	
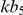	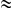	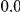	
**Degradation rate constants**			
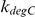	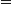	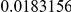	
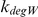	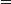	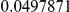	

Constant parameters used in the model. Reaction and degradation rate-constants are determined according to formulas applied to the free-energies and activation energies which are hand-designed to adhere to the constraints inherent in our definition of a minimal metabolism and to display the phenomena of interest.

The metabolism dynamics are simulated by numerical integration of the differential equations in Table 2 (we used an Euler timestep of 0.01 and typical chemical concentrations ranged between 0 and 2.0). These equations include some reactants that are only used in certain experimental scenarios and are explained later in the text. The rate constants (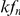 and 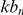) in the differential equations were determined by assigning *free-energies* to each reactant and *activation-energies* for each reaction such that the system adhered to the constraints given in our definition of a minimal metabolism. Given chemical free-energies and reaction activation-energies, reaction rates can be calculated by applying the following equations which indicate the reaction rate for a forward (exergonic) reactions and backward (endergonic) reactions respectively.
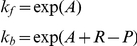



**Table 2:** Differential equations.

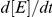	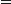	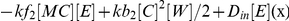
		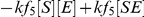
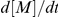	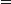	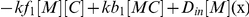
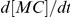	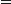	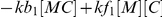
		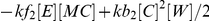
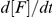	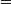	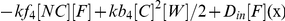
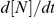	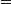	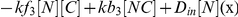
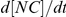	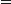	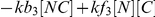
		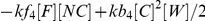
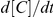	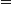	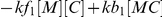
		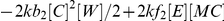
		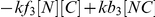
		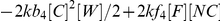 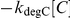
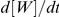	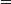	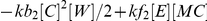
		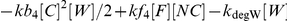
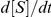	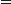	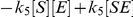
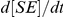	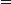	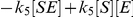

Full list of the differential equations that influence the change in chemical concentrations. Includes all chemical pathways used in the experiments. Parameters 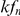 and 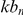 represent the reaction rate constants for the 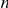
th reaction in the forward or backward direction. Those constants with ‘degK’ subscripts represent the rate of degradation of chemical K and constants with ‘
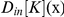
’ subscripts represent the concentration of resource 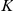 diffusing into the system from the environment at position 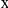. Table 1 indicates the values of the constants.

**Figure 4:**
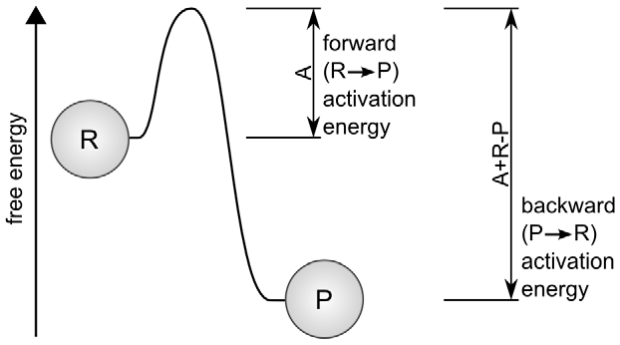
Energy required for a reaction to take place.

Figure 4 indicates why the forward and backward equations are different. This method of determining reaction rates allows the exploration of abstract chemistry while remaining congruent with the 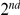 law of thermodynamics.

The environment of a bacterium can affect the concentration of certain chemicals within it, specifically: 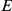, 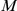, 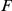, 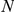, and 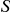. For simplicity, we assume that these resources are actively transported into the bacteria at a rate independent of the concentration of the chemicals inside the membrane. The internal resource levels are increased by continuous transport from the environment into the bacterium according to the following function:
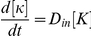
where 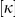 is the concentration of the relevant chemical inside the bacterium, 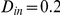 is the rate of transport across the membrane and 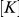 is the concentration of relevant chemical in the environment of the bacterium. This influence of the environment is included in the differential equations in Table 2 as the last terms of those equations that update chemicals that are affected by the environment (chemicals 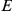, 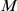, 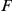, 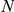, and 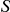).

### Implementation of movement

The above chemical reactions are simulated as enclosed within a membrane, comprising a simulated bacterium. Although minimal metabolisms have been the subject of simulation models, there have been very few attempts (see e.g., [46]) to study the dynamics of metabolism coupled to some form of movement generation mechanism. In this model, inspired by the motion mechanism of *E. coli* and other species, the simulated bacteria are capable of moving in either a directed, ‘running’ motion or by randomly changing their orientation (‘tumbling’). Bacteria are, by default, in a ‘running’ mode. Each iteration, however, a bacterium has a chance of tumbling that is proportional to the concentration of the product of the metabolism, 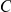: 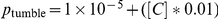. Running bacteria move in a straight line in the direction of their orientation (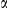), 
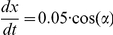
, 
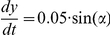
. Tumbling bacteria remain at the same location, with 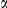 changed to a random value selected from a flat distribution between 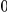 and 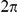.

**Figure 5:**
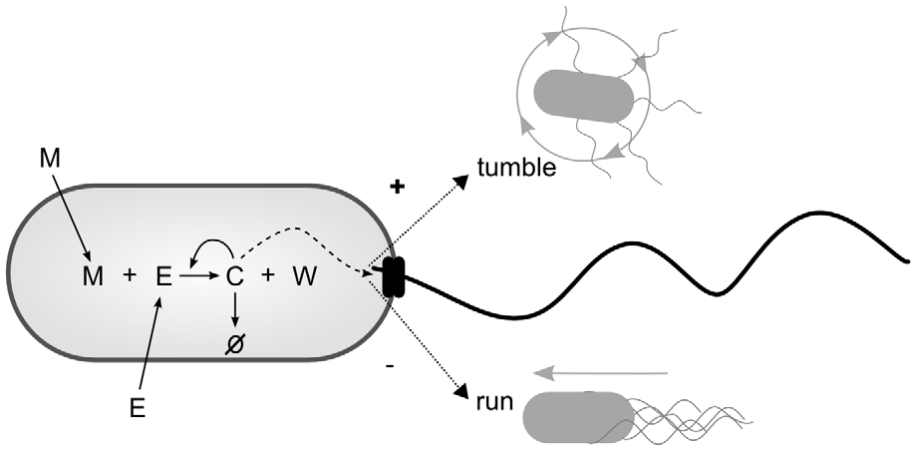
Diagram indicating the minimal metabolic reaction and its coupling to tumbling and running modes of behavior.

A full schematic diagram of the minimal metabolism and its coupling to behavioral mechanisms can be seen in Figure 5.

## Results

**Figure 6:**
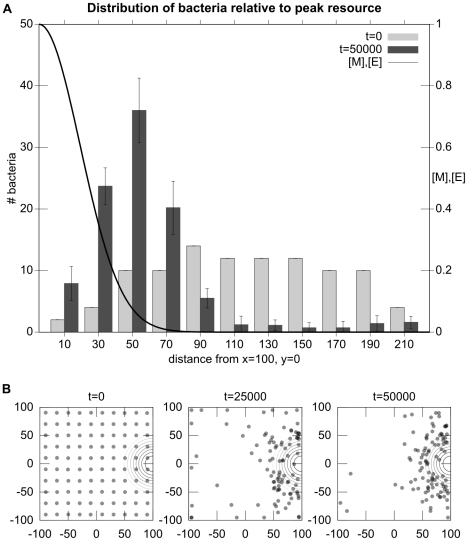
Experiment 1: Chemotaxis towards a gradient of 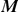 and 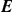. Plot A is a histogram that indicates the distance of bacteria from the location of highest concentration of 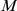 and 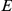 (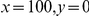) at the start and end of trials. Data are averaged from 10 runs of 100 bacteria each. Plot B indicates the spatial distribution of the simulated bacteria as time progresses in a typical trial. The concentric circles indicate the center of the Gaussian distribution of resources 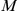 and 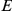.

We now describe five experimental scenarios where simulated bacteria are placed in environments containing different distributions of different chemicals compounds. At the start of each simulation, 100 simulated bacteria are distributed evenly around the 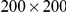 unit square environment in a 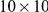 grid (indicated in the left-most plot of e.g. Figure 6B). Each iteration, the metabolism and position of each bacteria is updated according to the equations described earlier. Bacteria are all initiated with a low (0.05) concentration of their metabolites unless otherwise indicated. Except for Experiment 5, all environmental resources have a peak concentration of 1.0 and fall off with distance from their center according to the following equation where 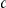 is the concentration of the relevant resources and 
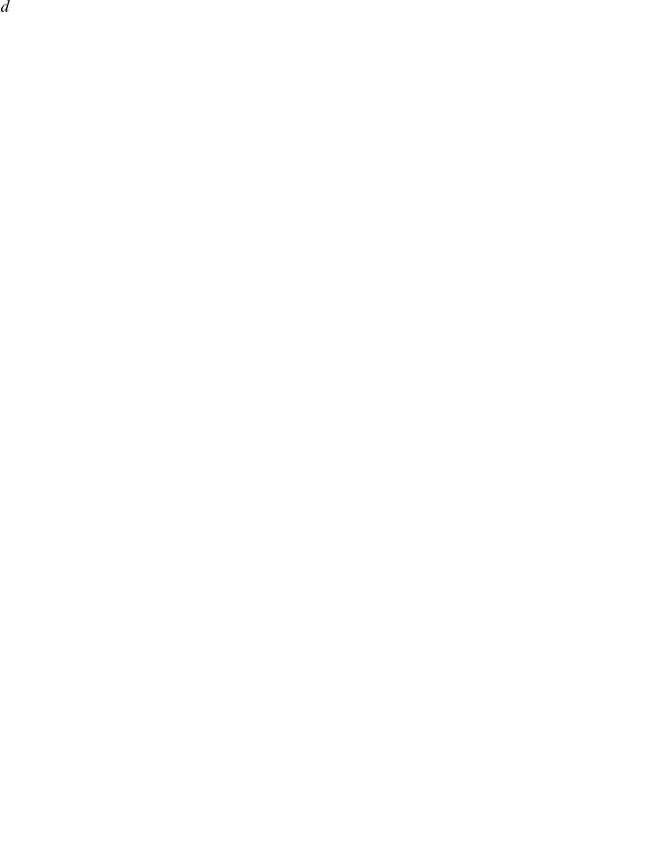
 is distance from the center: 
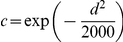
. Resources in the environment are always kept constant (i.e., there are no stigmergic effects – bacteria do not affect the concentration or distribution of resources in the environment).

### Experiment 1: Chemotaxis to metabolizable sources

In this first scenario, a source of 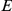 and 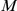 is centered at 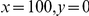. Figure 6 shows the distribution of simulated bacteria at the start, middle and end of a 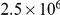 iteration simulation. It can be seen how bacteria perform chemotaxis to the area of high-concentration of 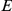 and 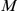 (which is indicated by the concentric circles).

When a bacterium has access to plenty of resources, it produces significant quantities of 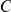 and 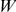. The high concentration of 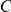 causes the rate of tumbling to increase to the point where the bacterium is more or less standing in place since the tumbling frequency is so high that it never runs for a significant distance in any direction. If, on the other hand, the bacterium has insufficient available resources to maintain high levels of 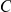, the probability of tumbling will fall and the bacterium will perform a combination of running and tumbling that results in a random walk. This random walk will continue until it comes across a region where it can produce sufficient 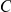 to push it “above threshold”. In this manner, the simulated bacteria perform a simple form of “selective stopping” chemotaxis whereby they move in a random manner until they are in a resource rich area, at which point they tend to remain where they are.

Statistically, the simulated bacteria show a correlation between final location of the bacteria and high concentration of metabolizable substrates in the environment, i.e., a “chemotactic” movement towards high-concentration of attractants. The individual behavior of the bacteria may not follow a direct chemotactic path, but the probabilistically directed (i.e., corrected or regulated) behavior clearly results in an up-gradient movement tendency. Note that even in experimentally observed chemotaxis in *E. coli*, with their more sophisticated gradient-climbing adaptive strategy, the path followed by a single bacteria is difficult to characterize as chemotactic, it is rather the global effect of a population of bacteria that results in a clear chemotactic distribution. This experiment demonstrates that a metabolism-based control of flagellar rotation could potentially perform chemotaxis without dedicated signal transduction pathways, transmembrane receptor proteins nor sensory adaptation.

### Experiment 2: Local presence of metabolizable resources inhibits chemotaxis to other resources

Alexandre and Zhulin observed that “the presence of another metabolizable chemical (exogenous or endogenous) prevents chemotaxis to all attractants studied” [21, p.4682]. We tested our simulated metabolism-based chemotactic bacteria to confirm that they also undergo inhibition of chemotaxis due to the presence of alternative metabolizable resources. To perform this test we used the above chemotaxis scenario as a control. The new experimental scenario is identical to the first except that two new resources, 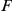 and 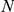, are included, uniformly distributed throughout the environment at a concentration of 0.5.

**Figure 7:**
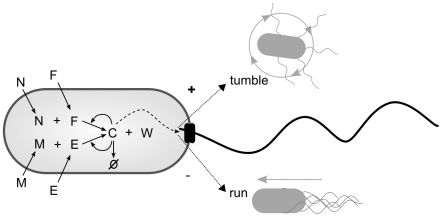
Diagram indicating metabolic reactions for Experiment 2.

**Figure 8:**
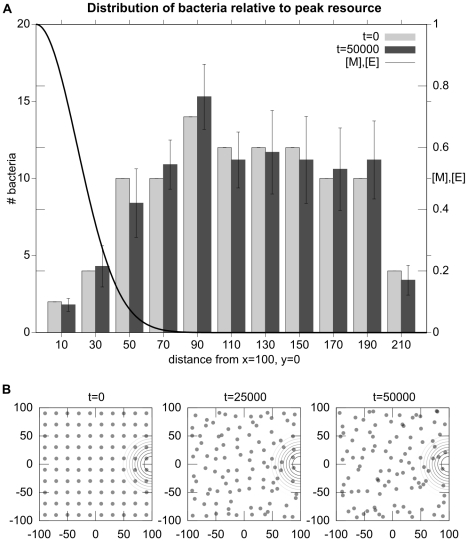
Experiment 2: Abundance of sufficient resources inhibits chemotaxis to attractants. In this trial, agents were placed on the same gradients as in Figure 6, but with an additional uniform distribution of alternative metabolizable sources 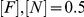. The presence of these resources clearly inhibits chemotaxis to 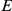 and 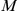.

For this experimental scenario (and subsequent ones) alternative resource molecules and reactions were required. For the sake of simplicity we created a duplicate metabolic pathway with identical stoichiometry, reaction rates, etc., the only difference being the chemicals involved (see Figure 7): resource 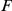 (as analogous to 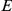) and material resource 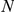 (analogous to 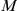).
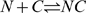
(3)

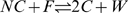
(4)The results can be seen qualitatively by comparing Figure 6 (the control) and Figure 8. It is clear that chemotaxis has been inhibited. The mean distance from the source (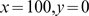) for 10 runs of 100 agents each was 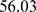 (std. 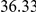) for the control, and 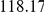 (std. 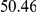) for the experimental abundance of alternative resource condition.

The mechanism for this inhibition is simple. Resources 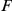 and 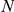 are ubiquitous and sufficient to maintain the concentration of 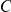 at the high value necessary to keep the bacteria tumbling. The predominant tumbling keeps the agents stationary, preventing any chemotaxis to the 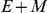 resource.

### Experiment 3: Inhibition of metabolism to a resource inhibits chemotaxis to and only to this resource

A second result was published by Alexandre and Zhulin in support of energy-taxis as the primary mechanism of chemotaxis in *A. brasilense*: “[The] inhibition of the metabolism of a chemical attractant completely abolishes chemotaxis to and only to this attractant” [21, p.4682].

**Figure 9:**
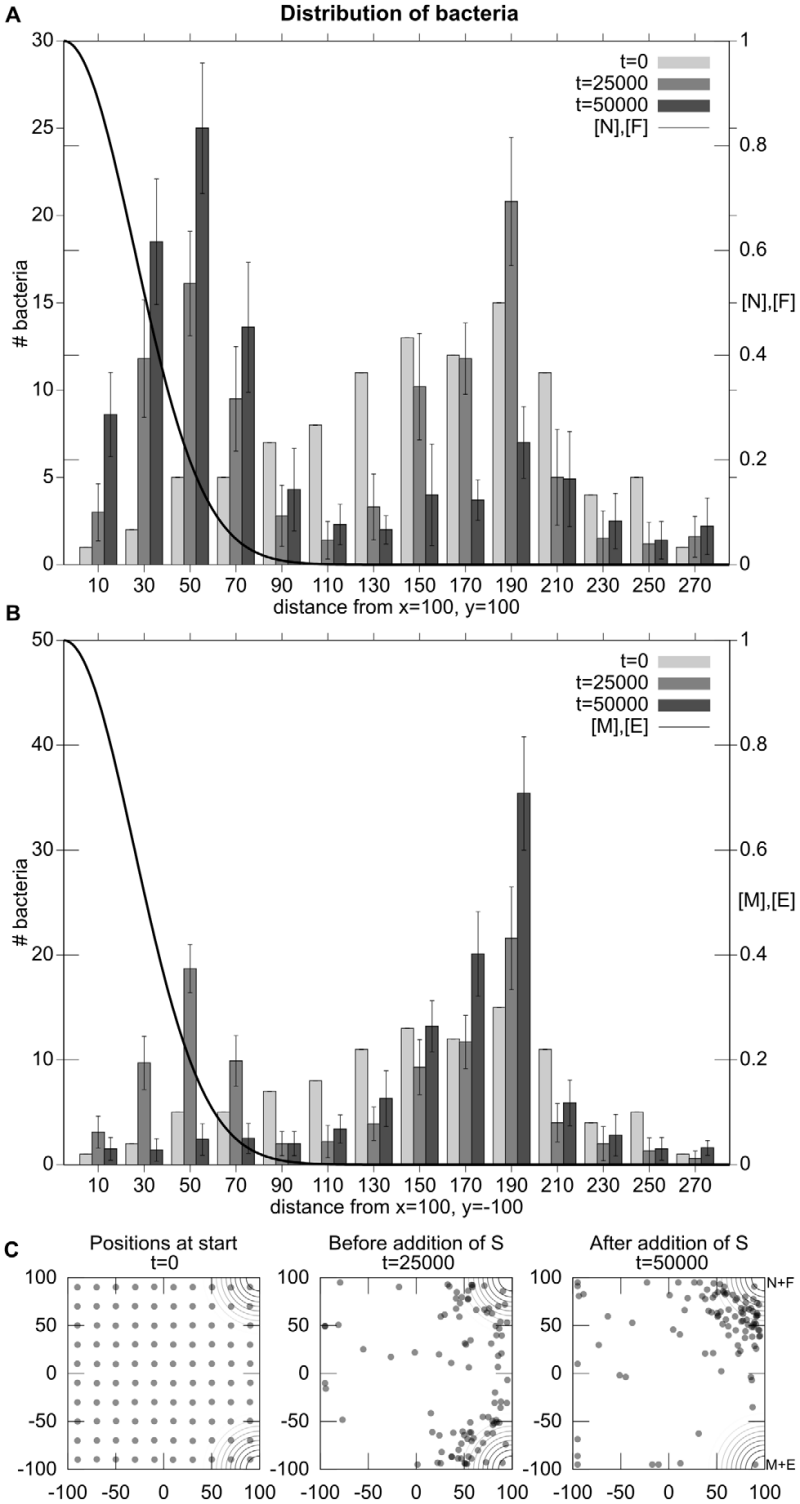
Experiment 3: Inhibition of a metabolic pathway inhibits chemotaxis only to the relevant resources. Placed in an environment with two sets of metabolizable resources (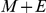 located in the lower-right and 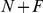 located in the upper-right), the simulated bacteria move from an initial, even-distribution to the areas higher in concentration of either resource-pair. The insertion of 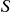 (a chemical that inhibits the metabolism of 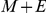) causes the simulated bacteria to cease chemotaxis towards the no-longer metabolizable resources without influencing chemotaxis to the other attractants. Histogram A illustrates average distance to the non-inhibited (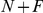) source. Histogram B shows the distance from the inhibited source (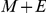). As before, data is taken from 10 trials of 100 bacteria each. Plot C illustrates the spatial distribution of simulated bacteria in a typical trial.

To test this phenomenon in our simulation of metabolism-based chemotaxis, simulated bacteria are placed in an environment with two resource gradients. The first, consisting of equal parts of resources 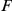 and 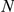 is highest in concentration in the upper-right corner of the simulated environment. The second is equal parts of 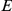 and 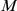 and is highest in concentration in the lower-right corner. In the upper-right corner, resources 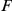 and 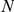 are sufficient for a healthy metabolism to continue to autocatalyze 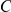 and maintain its concentration high enough for the bacteria to remain in this corner. The same is the case for 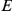 and 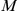 in the lower-right corner. This can be seen in the central bottom plot of Figure 9.

**Figure 10:**
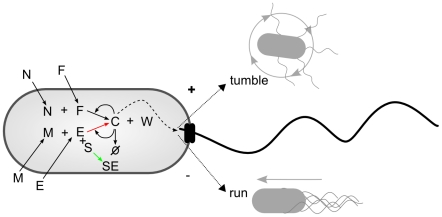
Diagram of the metabolic reactions for Experiment 3.

Halfway through this scenario, we add a uniform concentration of 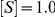 to the entire simulated environment. This chemical inhibits the 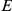/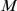 metabolic pathway by exothermically and rapidly bonding to metabolizable substrate 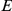, transforming it into a non-reactive chemical, 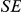 (see Figure 10). This process is described by the following reaction equation:
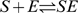
(5)


After 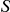 is added to the environment, the simulated bacteria cease to remain in the area high in concentration of 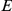 and 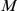, but continue to be attracted to the high concentrations of 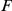 and 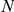, as shown in the right-most plot of Figure 9 (bottom), demonstrating inhibition of chemotaxis to a reactant by inhibition of metabolization of that reactant.

### Experiment 4: Metabolic inhibitors act as repellents

Specific metabolic inhibitors such as oxidized quinones or specific electron transport inhibiting molecules such as myxothiazol have been shown to inhibit chemotaxis [47]. It has also been shown that such metabolic inhibitors can act as repellents [19], [48]. We tested to see if our model could also display metabolic inhibitors (or toxins) acting as repellents.

**Figure 11:**
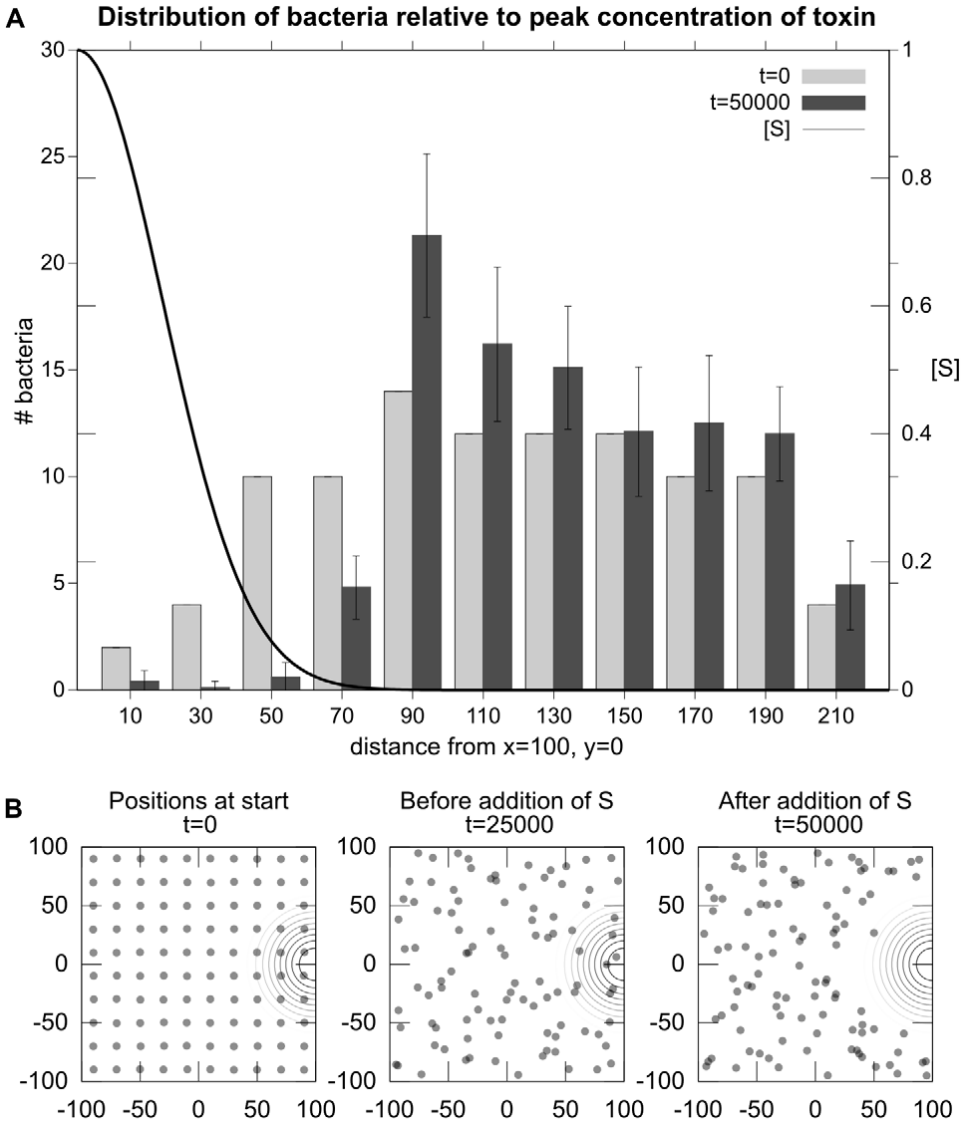
Experiment 4: Metabolic inhibitors act as repellents. In a uniform distribution of resources, simulated bacteria move away from high concentrations of the metabolic inhibitor 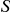.

**Figure 12:**
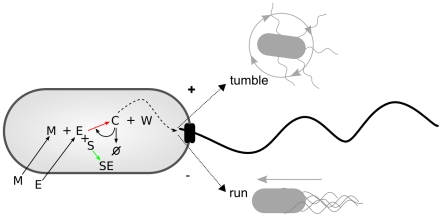
Diagram of the metabolic reactions for Experiment 4.

A repulsion due to a metabolic toxin can be clearly seen in Figure 11 which shows a scenario in which bacteria are evenly distributed in an environment of uniform distributions of 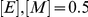. Halfway through the simulation, a gradient of metabolic inhibitor 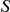 is added to environment (with a peak concentration 5.0, centered at 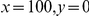) and the bacteria move away from the higher concentrations of that toxin. Figure 12 indicates the reactions that occur in this scenario.

### Experiment 5: Sensitivity to history

Experiments 1–4 have reproduced empirical observations made by Alexandre and Zhulin. The following experiments explore additional phenomena that could lead to some empirical predictions. This scenario is inspired by Alexandre and Zhulin's observation that bacteria demonstrate a sensitivity to their history of exposure to different resources. Specifically, “[s]tronger chemotaxis responses are observed when cells are grown on the sugar under test as the growth substrate” [21, p.4682]. In this experiment, there is no 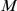 in the environment except along a strip defined by 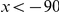. Agents are initialized with no 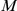. A gradient of 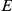 is placed at the center of the right side of the environment.

**Figure 13:**
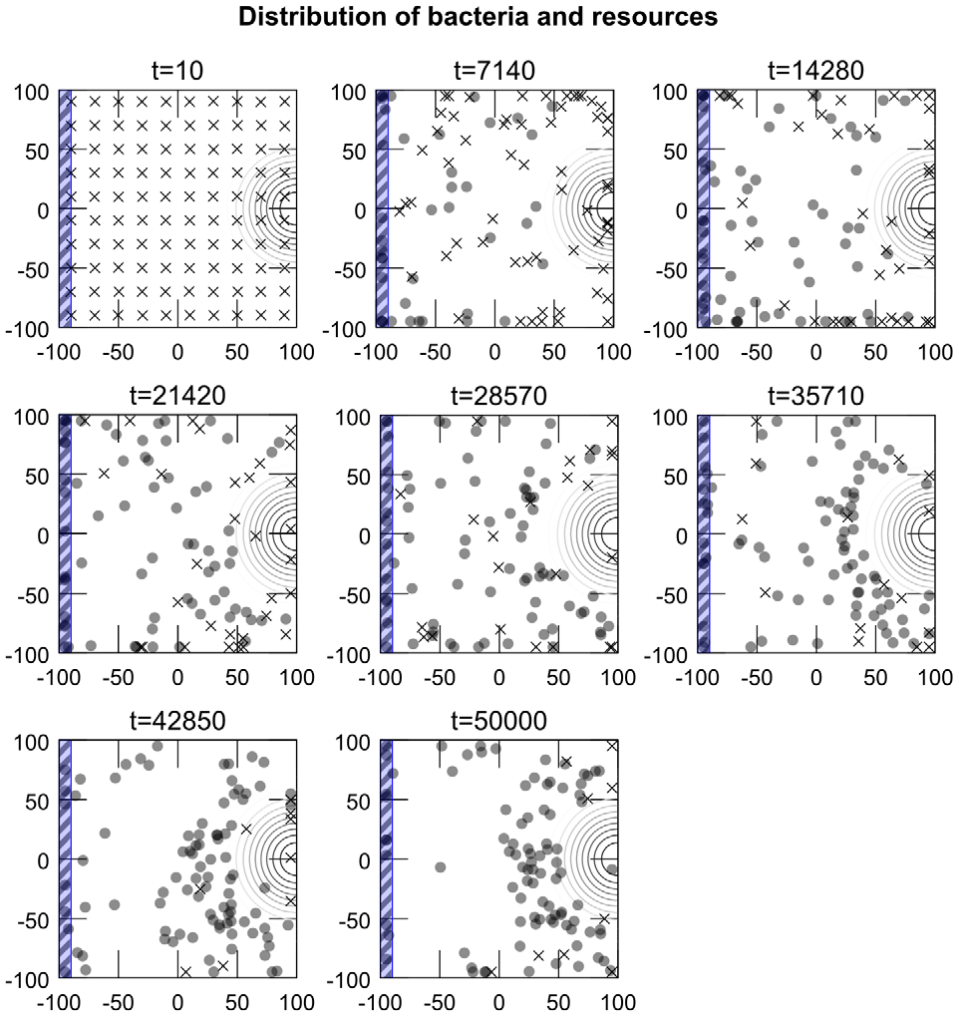
Experiment 5: Sensitivity to history. The metabolism of the bacteria changes when encountering 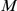 (located at the left side of the environment), as 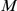 is incorporated into the cell, making 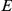 metabolizable. The 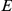 rich area (concentric circles on right side of plots) is initially insufficient to support production of 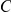, but for agents that have incorporated 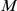 into their metabolism, 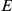 becomes an attractant. Bacteria with 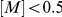 are shown as 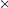
s rather than circles.

Bacteria have no 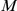, so the resource 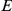 is insufficient to produce 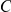. Only once they have encountered the region with 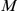 and incorporated 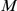 into their metabolism does 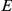 act as an attractant. This process can be seen through observation of Figure 13 where agents are drawn with 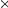
s if they have concentrations of 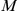 less than 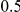 and as circles otherwise. Early in the simulation, agents tend to run as none have access to resources sufficient to produce and maintain significant quantities of 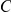. As time passes, the random motions of the bacteria cause some to encounter the 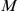 on the left. More time passes, and these agents, now rich in 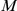 can produce 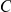 while in areas rich in 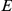. At this point, chemotaxis towards high concentrations of 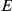 is observed.

The experiment shows how what becomes an attractant for metabolism-based chemotactic bacteria is not an environmental compound *per se*, but rather what, at a given point on the history and internal state of the bacterium, is required for metabolism to occur. Bacteria performing metabolism-based chemotaxis operate according to their current metabolic needs.

### Experiment 6: Integration of environmental phenomena

**Figure 14:**
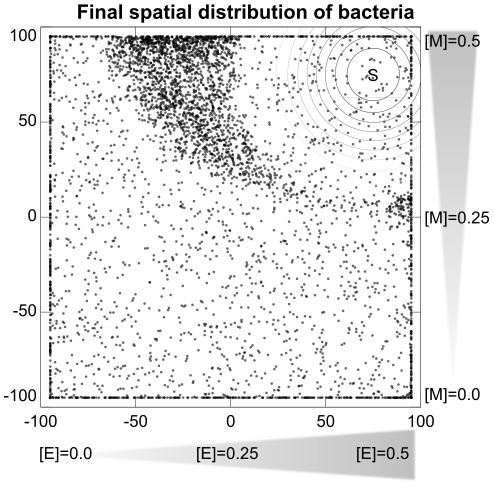
Experiment 6: Metabolism-based behavior does not respond directly to environmental features, but rather to their combined effect upon metabolism. Here we see simulated bacteria concentrated in the area of the environment where the combined influence of all environmental phenomena allow for a sustainable metabolism.

By basing chemotaxis in metabolism, the simulated bacteria respond not to specific environmental phenomena, but to the combined effects of all environmental features upon metabolism. In Experiment 6, we demonstrate this ability to integrate environmental phenomena by placing bacteria in a more complicated environment than those of previous experiments. The environment for Experiment 6 consists of perpendicular linear gradients of resources 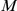 (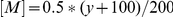) and 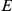 (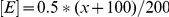) with a Gaussian distribution of toxin 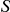, with a peak concentration of 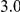 centered at 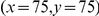. Figure 14 indicates the final position of 5000 bacteria (the results of 50 trials, each with 100 evenly distributed bacteria as in the other experiments). It can be clearly seen that the bacteria are neither maximizing concentrations of 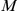 or 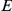, nor the combination of them, but are performing chemotaxis to the areas where the combined effects of the environmental resources 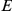, 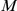, and 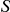 allow the metabolism to operate sufficiently well. The overall bacterial distribution appears correlated with the spatial distribution of the optimal combination of compounds.

One interesting aspect of this plot is the asymmetric distribution of bacteria along the x and y axes, corresponding to the gradients of 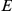 and 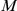 respectively. It appears that for our model bacteria, it is more important to have a high concentration of 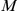 than of 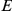. This difference may be caused by a high concentration of 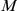 transforming ‘free’ 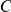 into 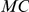. 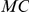, unlike 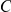, does not degrade, so a high concentration of 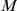 makes the metabolism less likely to degrade than a high concentration of 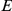. An alternative possibility is that the bottleneck in the metabolism lies in the first reaction (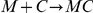) as this reaction has a slower rate constant than the second reaction (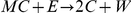). High 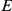 has little influence on this bottleneck, but a significant concentration of 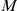 can open up the bottleneck, allowing for a more rapid production of 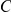. We confirmed that a third possibility, the asymmetric influence of 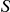 upon the reactions, is not responsible for the asymmetric distribution of bacteria (results not shown).

It is interesting to note that in aerotaxis experiments with e.g. *E. coli* and *A. brasilense*, the aerotactic bands can form an asymmetric profile as well (see e.g. [49, p.2238 Figure B, top]). The environmental conditions at the different sides of these bands could be slightly different with respect to the metabolism of the bacteria, and perhaps a mechanism similar to that described here could explain the asymmetric distribution of bacteria in these experiments.

## Discussion

Research in bacterial chemotaxis has operated largely under the assumption that the behavior is supported by transmembrane receptors and dedicated signalling pathways and that such pathways are metabolism-independent. Despite the growing body of evidence that in many species this might not always be the case (even for those largely thought to be so, such as *E. coli*) available simulation models of bacterial chemotaxis assume metabolism-independence. Here we have presented the first minimal model of metabolism-based chemotaxis. It recreates phenomena observed in bacteria and allows us to explore some potential consequences of metabolism-based behavior.

The behavioral strategy employed by the simulated bacteria in our model is the “selective stopping strategy” in which bacteria move around in a random walk until they reach a satisfactory area, at which point they tumble in place. Recent artificial evolution of simulated chemotaxis [26] has shown that this strategy (also referred to as the “non-adaptive or inverted response”) is, under certain ecological constraints, the most likely chemotactic strategy. It has also been observed in some cases of metabolism-dependent chemotaxis [34]. In order to address how metabolism could directly produce such behavioral patterns, we have developed a model of what we have called *metabolism-based chemotaxis* (a simpler case than that of metabolism-dependent chemotaxis). We first identified a minimal metabolic organization as that of an autocatalytic reaction. We then assigned a probability of running or tumbling to the concentration of the auto-catalyst. The resulting system is very simple, yet capable of instantiating four chemotactic phenomena observed in bacteria.

- Chemotaxis to metabolizable compounds. [Experiment 1]
- A local abundance of metabolic resources inhibits chemotaxis to other resources. [Experiment 2]
- Inhibition of the metabolism of an attractant inhibits chemotaxis to that attractant, and that attractant alone. [Experiment 3]
- Metabolic inhibitors act as repellents [Experiment 4]

The observations of history dependence reported by Alexandre and Zhulin led to the exploration of Experiment 5 where a compound is incorporated into metabolism and results in a change in chemotaxis pattern according to past experience.

Finally, Experiment 6 demonstrated the capacity of metabolism-based chemotaxis to respond appropriately to combinations of a variety of simultaneous environmental influences. This experiment showed the potential of metabolism as a mechanism for effective chemotactic integration.

The present simulation is not a model of the specific mechanisms supporting metabolism-dependent chemotaxis in bacteria. Yet, it serves as a proof of concept of how a very simple abstraction of metabolism can support, without the addition of specific signaling pathways and even without the need of transmembrane receptors, a wide range of chemotactic phenomena. As a conceptual model it can be further used to explore some theoretical implications of relaxing the metabolism-independent assumption.

Evidence has been found of metabolism-dependent chemotaxis where metabolic processes such as the electron transport system influence flagellar rotation indirectly, by way of the dedicated chemotactic two-component signaling system. There is also evidence for a mechanism through which metabolism directly influences flagellar rotation, i.e., without an intermediate dedicated signaling system, in a manner more similar to the metabolism-based chemotaxis modeled here. Specifically, it has been shown that *E. coli* can perform chemotaxis even when stripped of most of the signaling pathway typically associated with chemotaxis [35], suggesting that there might be at least two different mechanisms supporting chemotaxis in *E. coli* [2, p.575]. Interestingly, the concentration of fumarate, an intermediate in the citric acid cycle that is part of the “universal metabolism” [50], has been shown to influence the direction of flagellar rotation. A high concentration of this metabolic product increases chance of clockwise, tumble inducing, rotation; the same relationship of metabolic influence upon flagellar rotation that is used in the selective-stopping strategy. Fumarate operates directly upon the flagellar motor switch [51] in a manner that is independent of the protein signaling pathway typically associated with chemotaxis [52]. It turns out that fumarate might be currently instantiating mechanisms of metabolism-based chemotaxis; something that still remains to be experimentally tested. This hypothesis was anticipated by [26, p.5] and we have shown how fumarate-like intermediate metabolites (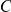 in our model) could not only produce simple chemotaxis but could reproduce a wide spectrum of non-trivial chemotactic phenomena. What is needed to achieve these behavioral patterns is not a complex system of transmembrane receptors influenced by metabolism in subtle ways but simply a metabolite capable of influencing flagellar rotation in the right manner.

The present model also suggests that it might be time to re-consider part of the terminology and the externalist approach to chemotactic studies. For instance, it is generally assumed that environmental compounds are invariably either *attractants* or *repellents* for bacteria, as if bacteria were simply stimulus-driven systems. The model of metabolism-based chemotaxis shows, however, that environmental compounds are not attractants or repellents purely on the basis of their binding properties and their stereotypically elicited responses. Environmental compounds must instead be categorized within the context of metabolism, which is influenced by the history of the cell and its internal organization (metabolic rates, active and non-active metabolic pathways, etc.). In other words, the behavioral significance of chemical compounds becomes a relational property that depends on the metabolic dynamics of the cell (which cannot be abstracted away in the study of behavior). As Experiment 3 shows, if a resource ceases to be metabolized, it ceases to act as an attractant for bacteria. Also, (as shown in Experiment 4) chemical compounds that are toxic for the metabolism of the bacteria can act as repellents without the need of any specific binding of it, or even without the bacteria ever encountering that compound in its evolutionary past. This capacity to be behaviorally sensitive to the effects of environmental compounds on metabolism provides a powerful means of behavioral evaluation and increased adaptive response (at the organismic level). It is not clear how the same adaptation could occur for a metabolism-independent mechanism that requires binding with specific compounds to elicit specific responses.

While advances have been made on the understanding of how receptor complexes integrate sensory information [53], [54] the potential *integrative role of metabolism* remains under-explored. The classic view is that integration in metabolism-independent chemotaxis is accomplished in the group dynamics of the transmembrane sensors that all modulate CheA activity [55]. This metabolism-independent mechanism of integration relies upon specific interactions between stimulus chemicals and transmembrane sensors. We can compare this to metabolism-based behavior, which responds not directly to environmental phenomena but to the *combined effects of environmental phenomena upon the metabolism*. This indirect sensitivity to the environment makes it a good candidate for integrating different stimuli and producing the appropriate response (move toward or away). Goldstein and Soyer [26] acknowledge this issue but their model does not address any integrative phenomena—their simulation results correspond only to a single attractant gradient scenarios. Despite its simplicity, the model presented here is able to effectively integrate information from multiple gradients in a straightforward manner (Experiment 6). By being sensitive to the production of 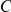, it integrates the effect of all environmental features upon metabolic rate.

As an example of the potential integrative power of metabolism-based chemotaxis, imagine two compounds, 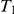 and 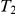, each of which acts as a metabolic toxin when encountered on its own. But, when encountered together, they act as excellent metabolic resources. Metabolism-based chemotaxis would respond appropriately (move towards 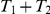 when encountered together and away from 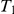 or 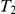 when either is encountered on its own), while metabolism-independent chemotaxis would require the evolution of considerable specific machinery to accomplish the same appropriate behaviors.

A further development of the model presented here (see [56]) has allowed us to explore the potential of metabolism-based mechanisms to produce gradient-climbing strategies, in particular we have shown how a single new reactant could turn a network of metabolic reactions that produces the selective-stopping behavior into one that produces the more intricate gradient-climbing behavior. We have also designed a scenario where a simulated protocell incorporates a new attractant from the environment into its metabolism and becomes chemotactic towards it. We have used the above experiments to explore theoretically the potential of metabolism-based chemotaxis, the feedback between metabolism and behavior, to bootstrap and accelerate early evolutionary processes [56].

Among the further extensions, a very interesting development would be to study which situations are conducive to which relationships between metabolism and chemotaxis and how transitions from one form to the other could occur. In particular, artificial co-evolution of metabolic networks and behavioral mechanisms could help address questions regarding a) the likelihood of metabolism-dependent or metabolism-independent chemotaxis under various environmental conditions, b) the possibility of metabolism-independent chemotaxis arising from metabolism-dependent precursors and c) how both types of chemotaxis might co-exist with a varying degree of influence.

Despite the considerable advances that the segregated one-compound-one-response approaches to chemotaxis have provided so far, it is perhaps time to start integrating not only metabolism into the picture but richer and varying environments where metabolic modulation might be playing a more relevant behavioral role. We hope to have shown that such an integrative move does not necessarily require the inclusion of an overwhelming level of detail, but might instead be effectively dealt with by metabolism-based forms of regulation. Many aspects of metabolism can potentially be abstracted away to reproduce the higher order dynamics of complex metabolic networks and then coupled to a behavioral mechanism. Moving in this direction opens the space for interactions between internal and environmental chemical dynamics that are not reducible to the influence of environmental compounds upon transmembrane receptors.

From the reported experiments we can generalize that, despite its simplicity, metabolism-based chemotaxis allows for an *ongoing* evaluation of environmental conditions. This evaluation is *indirect* in that behavior is not in response to the environment, but rather to the influence of the environment upon the metabolism. The ongoing and indirect nature of metabolism-based chemotaxis makes possible an automatic and appropriate response to a variety of encounters with environmental conditions that have never been experienced by the bacterium, nor even by its evolutionary ancestors, for it is not necessary to evolve trans-membrane sensors that interact in specific ways with each environmental influence. The evaluation of the environment is accomplished by the influence of the metabolism.

These generalizations should be further examined both by empirical studies and elaborations of the current model. We would like to stress that the current model plays the role of a proof of concept by allowing us to see the possibility of metabolism-based chemotaxis at work and unveil some implications. As variations of the model start to address more specific issues, they will have to incorporate more realistic assumptions such as energetic requirements for movement, biomechanics, differences in timescales between behavior and metabolism, and potential interactions between optimal behavioral control, metabolic dynamics and stochasticity. Also required is a study of the parametrical robustness of the phenomena reported here.
